# Semi-Supervised Cross-Subject Emotion Recognition Based on Stacked Denoising Autoencoder Architecture Using a Fusion of Multi-Modal Physiological Signals

**DOI:** 10.3390/e24050577

**Published:** 2022-04-20

**Authors:** Junhai Luo, Yuxin Tian, Hang Yu, Yu Chen, Man Wu

**Affiliations:** School of Information and Communication Engineering, University of Electronic Science and Technology of China, Chengdu 610056, China; 202021011418@std.uestc.edu.cn (Y.T.); 202121011427@std.uestc.edu.cn (H.Y.); 202022011429@std.uestc.edu.cn (Y.C.); 201821011420@std.uestc.edu.cn (M.W.)

**Keywords:** DEAP dataset, electroencephalogram (EEG), emotion recognition, multi-source fusion, stacked denoising autoencoder, unsupervised representation learning

## Abstract

In recent decades, emotion recognition has received considerable attention. As more enthusiasm has shifted to the physiological pattern, a wide range of elaborate physiological emotion data features come up and are combined with various classifying models to detect one’s emotional states. To circumvent the labor of artificially designing features, we propose to acquire affective and robust representations automatically through the Stacked Denoising Autoencoder (SDA) architecture with unsupervised pre-training, followed by supervised fine-tuning. In this paper, we compare the performances of different features and models through three binary classification tasks based on the Valence-Arousal-Dominance (VAD) affection model. Decision fusion and feature fusion of electroencephalogram (EEG) and peripheral signals are performed on hand-engineered features; data-level fusion is performed on deep-learning methods. It turns out that the fusion data perform better than the two modalities. To take advantage of deep-learning algorithms, we augment the original data and feed it directly into our training model. We use two deep architectures and another generative stacked semi-supervised architecture as references for comparison to test the method’s practical effects. The results reveal that our scheme slightly outperforms the other three deep feature extractors and surpasses the state-of-the-art of hand-engineered features.

## 1. Introduction

Objective affective analysis has always been in the area of psychology over the course of the twentieth century. It is mainly in the form of psychologists determining treatment options based on patients’ self-reported emotional states. However, since the beginning of the 21st century, with the rise of artificial intelligence, emotional research has broken into the field of computer science and information technology with the new name of “Affective Computing” [1], attempting to provide “Emotional Intelligence”, the almost universally acknowledged gap between artificial intelligence and real machine intelligence [1,2,3,4]. Meanwhile, researchers from the fields of ethology, behaviorists, anthropology, etc., have also explored different aspects of this with various research purposes. Hence, in recent decades, effective research has been naturally interdisciplinary, pulling together knowledge from neurobiology, psychology, evolutionary biology, computer science, and beyond. Note that in this piece, “emotion” and “affection” are utilized to represent the same notion, but both are strictly distinguished from terms like “mood”, “feelings”, “attitude”, “personality”, etc.

Human emotions can be expressed through both verbal cues and nonverbal cues. The majority of pilot studies in the community used verbal expressions, particularly those derived from audio, images, and videos. Since affection is a highly personal property, emotions may be inhibited and elaborately cloaked in subjective verbal reports that vary from subtle hints to explicit declarations. Besides, language itself introduces ambiguity. However, nonverbal cues are spontaneous physiological changes in the process of experiencing waves of emotions, such as facial expressions, tone of voice, overt actions, slips of the tongue, and physiological voltage fluctuations (e.g., electroencephalogram (EEG), galvanic skin Response (GSR), electromyography (EMG), respiration, and temperature) from peripheral/central nervous systems more recently. It was reported in [5] that the Japanese would disguise negative facial responses with smiles in the presence of a scientist. By contrast, spontaneous expression of physiological fluctuations is less likely to be spurious. Consequently, the research community has started to focus its attention on modalities of genuinely physiological or the fusion of multiple modalities, including video, speech, and physiological recordings. To stay current with academic trends, in this article, we choose to stay focused on the pure physiological fluctuations elicited by experiencing various emotions captured by sensors on the body (EGG).

The central purpose of affective analysis is emotion recognition or emotion detection. Literature in this area can be divided into two categories, i.e., traditional machine-learning algorithms (can process raw data, with less accuracy) that depend on the most discriminating features extracted and selected by labor-intensive intervention and deep-learning algorithms that can work well on raw data.

Traditional machine-learning models such as support vector machine (SVM), decision trees, k-nearest neighbors (KNN), logistic regression, linear discriminant analysis, and dynamic time warping (DTW) have been utilized on various data in many applications. They are usually combined with manual feature engineering to extract the optimal robust feature set. Besides, a discrete hidden Markov model (HMM) was utilized in [6] as the classifier, and a hierarchical binary decision tree approach was proposed in [7], both of which were efforts in speech signals-based emotion recognition. Databases for the analysis of spontaneous emotions include MAHNOB-HCI [8] and DEAP [9]. Data in MAHNOB-HCI and DEAP ware both recorded with the Valence-Arousal-Dominance model. Most references used DEAP as their dataset. One study [10] used the Gaussian Process Latent Variable Model (GP-LVM) and applied SVM as its classifier on the dataset DEAP. The results showed that the accuracy of third-class classification was around 88/90% by considering emotions: VA.

In representation learning, deep generative-learning models like Deep Belief Network (DBN), Autoencoder (AE), and Variational Automatic Encoder (VAE) are employed to extract the hidden, abstract, and complex non-linear patterns of raw data, replacing or eliminating the hand-crafted steps. In addition, deep discriminative architectures such as Deep Neural Network (DNN) and Convolutional Neural Network (CNN) can also be regarded as feature extractors that learn hierarchical representations. For instance, CNN is believed to be capable of capturing spatial features (e.g., local dependencies) and invariant features in data. Another study [11] conducted the experiment on the dataset DEAP, and used DNN and CNN as classifiers, splitting the 8094 datapoints into ten batches, and gained a relatively high accuracy by considering emotions: VA. Long Short-Term Memory Network (LSTM), the building block of recurrent neural networks, can be used to exploit the temporal dynamics in time-series data.

Meanwhile, some papers had their own datasets. For example, N. Murali Krishna et al. tested six mentally impaired subjects using an eight-channel EEG signal to collect audio-visual stimuli. The features were extracted by cepstral coefficients with a novel generalized mixture distribution model. By considering four emotions: happy, sad, neutral, and boredom, the average resulting accuracy was about 89% [12]. H. Becker et al. also started their own dataset, HR-EEG, which included 257-channel EEG data. They considered various feature extractors, and used ANN as their model. Finally, they compared the influence of nine features and the accuracy of the experiment was medium [13].

We compare the emotion estimation methods we mentioned above in Table 1. We can see that although the accuracy of our method is not the highest, we have the highest number of features, and thus, the most comprehensive list for feature importance (occurrence frequency). Besides, we innovatively used SDA as a feature extractor and compared the proposed SDA with other networks with a best-performance assessment.

In this paper, we aim to construct a deep-learning algorithm with an SDA architecture including unsupervised pretraining and supervised fine-tuning using backpropagation of error derivatives that can process raw data with a relatively higher accuracy. We are motivated by the need to develop an efficient deep architecture to extract more powerful and robust representations automatically, to guide the learning of supervised learning algorithms. The top issue in emotion detection is the size of databases. More powerful representations are expected to be built with a larger scale of unsupervised learning. In the presence of labeled data and an abundance of unlabeled data, semi-supervised learning is more practical. In detail, the principal contributions of this paper are as follows:

1. We propose a semi-supervised deep-learning architecture to extract the underlying representations of pure physiological emotion data without any manual intervention. The experimental results indicate that the Stacked Denoising Autoencoder (SDA) ranks over the other three deep networks. To the best of our knowledge, ours is the first to utilize the stacked denoising autoencoder algorithm as the feature extractor and, in a true sense, to directly take the raw data as input for a model.
2. By looking back at the historical roots and the development of the emotion-recognition field, we implement most of the recommended well-designed features and perform a comprehensive comparative analysis of diverse existing methodologies and features on the same benchmarking database, thus proving that features from the wavelet transform are the most efficient.
3. The fusion of two different physiological modalities is performed on a data-level, feature-level, and decision-level. The results demonstrate that the two modalities operate simultaneously, instead of “EEG first, and periphery second”, as much of the literature claims.
4. Cross-subject and single-subject accuracy show a significant gap, which echoes the conclusions of previous studies in psychobiology (emotions are individualized). This gap can be reduced as the amount of data grows. To our knowledge, ours is the first to investigate the difference in performance between cross-subject and single-subject recognition.

The remainder of this paper is organized as follows. The derivation and selection of discriminating features are discussed in Section 2. Section 3 presents the premier well-performed recognizers in the literature. The experiment setup and result anlysis are presented in Section 4. Finally, Section 5 concludes this paper and discusses future work.

### Notation

Throughout this paper, the following notation is used: The EEG trace or the periphery physiological signal within a certain epoch is expressed as a function of time $\xi \left(t\right)\in R^{T}$, and T is the number of the time-samples in ξ. The time derivative of ξ(t) is denoted as $\dot{\xi }\left(t\right)$.

## 2. Material

As mentioned above, the benchmarking dataset, DEAP [9], is selected to compare the performances of different representations and algorithms. The physiological data in DEAP is adjusted differently to adapt to various algorithms, including a naive way of augmenting the limited samples to take advantage of deep-learning models and reducing the dimensionality by extracting the artificially designed features and the Principal Component Analysis.

### 2.1. The DEAP Dataset

Mathematically modeling emotion to represent affection’s latent structure is the first step in emotion-related research. Among the more popular theoretical frameworks for measuring emotion are the discrete model [5,14] and the dimensional theory. A comprehensive analysis of dimensional and discrete conceptual views on emotions has been completed by Eddie Harmon Jones et al. in their review [9], in which both models were believed to have much to offer in attempting to understand the psychology of emotions. From more recent literature, an obvious preference for a dimensional model exists in building physiological emotion databases, and it is indeed quite widespread today, gaining increasing acceptance. For example, the dataset MAHNOB-HCI [8] was recorded using the Valence-Arousal-Dominance (VAD) model; dataset DEAP [9] was also recoded along the scale of VAD. As a result, we also used the well-known VAD affection model to conduct the experiments.

The DEAP was a multimodal dataset for the analysis of human affective states. It contained the recordings of the EEG traces and 13 other periphery physiological signals of 32 subjects (half male) watching 40 one-minute music clips. Specifically, for one subject, there are 40 clips/trials. For one trial, 40 channels captured the physiological voltage fluctuations from where 45 electrodes were placed, resulting in 8064 time samples in a 60 s video time and a 3 s baseline with a 128 Hz sampling frequency for each channel considering that the inner affective states change slowly. We only use the data in the last 30 s because we need dimensionality reduction. The threshold of splitting a dimension of the affectioned model is simply set as 4.5, right in the middle of the range (0, 9), and thus obtaining the ground truth labels for the three basic recognition tasks (arousal, valence, and dominance).

**Figure 1:**
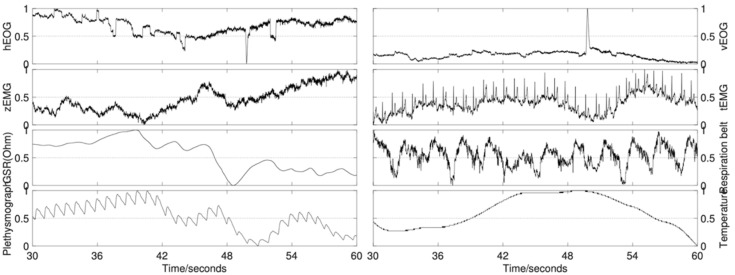
Illustration of signals from the eight periphery physiological channels in the last 30 s during a trial.

**Figure 2:**
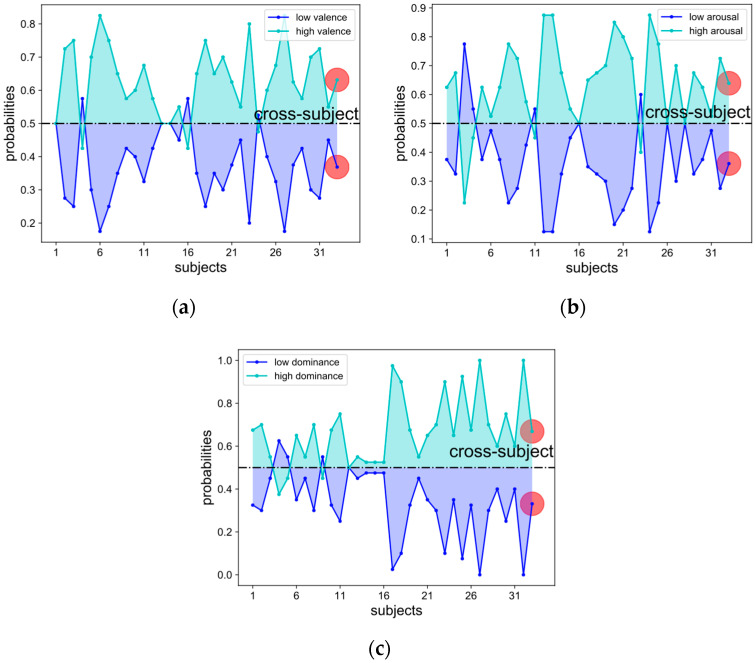
Prior probabilities of 32 single-subject recognition tasks and the cross-subject recognition (marked by the red dot) on all three scales of the dimensional effect model: (**a**) valence; (**b**) arousal; (**c**) dominance.

For the detailed placement of 45 electrodes, refer to [9] or their website. The 13 periphery signals are combined into eight channels for the five channels of horizontal electrooculogram (EOG), vertical EOG, zygomaticus major electromyography (EMG), trapezius EMG, and GSR derived from the difference between two electrodes. Normalized values of the eight periphery channels are illustrated in Figure 1. Prior probabilities of 32 single-subject recognition tasks and the cross-subject recognition (marked by the red dot) on all three scales of the dimensional effect model are illustrated in Figure 2.

### 2.2. Augmenting the Raw Data

Existing emotional databases share the same weakness: insufficient size. In this regard, datasets of visual and vocal modalities are no exception. For cross-subject emotion recognition, DEAP holds at most 1280 pieces of multimodal physiological data and corresponding subjective ratings on five dimensions of valence, arousal, dominance, liking, and familiarity, ranging from 0 to 9 continuously. The number 1280 is too few to fully exploit the power of deep-learning algorithms. In addition, even without considering the insufficient data volume, directly concatenating the overall 40 channels would result in a vector with 153,600 components (3840 × 40), where the three-second baseline is removed, and only the second half of the data is employed. As a result, 1280 pieces of input vectors of a length of 153,600 would lead to a shallow but wide network to avoid preventing overfitting. Therefore, some deep-learning pioneers in physiological emotion detection have tried to obtain smaller and more acceptable compressed representations from raw data.

However, deep-learning is desirable and valuable because it requires input of the original data rather than the extracted or compressed representations in addition to pre-processing (such as patching the missing values and normalization to eliminate magnitude differences). Ingeniously, we propose to consider signals captured from each channel as a separate piece of data. This is so that all channels of the same trial take the same subject evaluation scores accordingly. This simple augmentation will arrive at 40,960 pieces of data for EEG signals, 10,240 for periphery signals, 51,200 for data-level fusion, and 3840 components for each piece under the same conditions as above. The augmented raw data were normalized along the channel and then fed into our SDA network for feature extraction and classification.

### 2.3. Human-Designed Features

**Figure 3:**
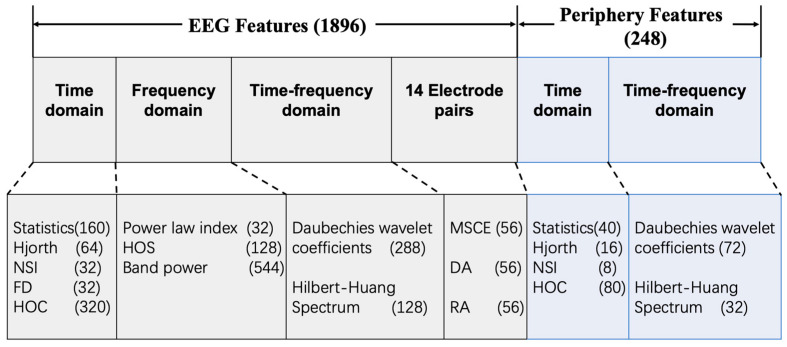
The specific dimensions of human-designed features implemented.

Pattern recognition algorithms are typically a combination of various features and learning algorithms. Generally, the pre-processed raw data were hard to process directly due to its large volume and the inabilities of classifiers to extract discriminative information from the data. Feature engineering is an essential way of taking advantage of prior knowledge and human ingenuity to compensate for the weakness of current learning models. Therefore, we must resort to features that characterize the most crucial information in raw signals. In emotion recognition, frequently utilized human-designed features can be classified into three categories: statistics derived from the time domain; the frequency domain; and the time–frequency domain. As illustrated in Figure 3 and Table 2 a total of 1896 EEG features and 248 periphery features were extracted.

#### 2.3.1. Time Domain

- Statistics of signals

The most common quantization terms are calculated entirely in the time domain, such as power reflecting the energy of the signal, the average over the whole-time interval representing the approximation with the lowest resolution in discrete wavelet transform, the variance that characterizes the degree of deviation, and difference (the approximate derivative of the discrete-time series). The power, mean, standard deviation, normalized 1st difference, and normalized 2nd difference of the 40 channels of one trial are computed.

(1) $$\mathrm{Power}:\;P_{\xi }=\frac{1}{T}\sum_{t=1}^{T}|\xi \left(t\right){|}^{2}$$

(2) $$\mathrm{Mean}:\;\mu_{\xi }=\frac{1}{T}\sum_{t=1}^{T}\xi \left(t\right)$$

(3) $$\mathrm{Standard}\;\mathrm{deviation}:\;\sigma_{\xi }=\sqrt{\frac{1}{T-1}\sum_{t=1}^{T}{\left(\xi \left(t\right)-\mu_{\xi }\right)}^{2}}$$

(4) $$1\mathrm{st}\;\mathrm{difference}:\;\delta_{\xi }=\frac{1}{T-1}\sum_{t=1}^{T}\left|\xi \left(t+1\right)-\xi \left(t\right)\right|$$

(5) $$\mathrm{Normalized}\;1\mathrm{st}\;\mathrm{difference}:\;{\bar{\delta }}_{\xi }=\frac{\delta_{\xi }}{\sigma_{\xi }}$$

(6) $$2\mathrm{nd}\;\mathrm{difference}:\;\gamma_{\xi }=\frac{1}{T-2}\sum_{t=1}^{T-2}\left|\xi \left(t+2\right)-\xi \left(t\right)\right|$$

(7) $$\mathrm{Normalized}\;2\mathrm{nd}\;\mathrm{difference}:\;{\bar{\gamma }}_{\xi }=\frac{\gamma_{\xi }}{\sigma_{\xi }}$$

- Hjorth parameter

The Hjorth parameter can be expressed as the activity, mobility, and complexity of a feature. The activity is the variance that can be fully expressed as the square of the standard deviation, so it will not be implemented afterward [15].

(8) $$\mathrm{Activity}:\;A_{\xi }=\frac{1}{T}\sum_{t=1}^{T}{\left(\xi \left(t\right)-\mu_{\xi }\right)}^{2}\;$$

(9) $$\mathrm{Mobility}:\;M_{\xi }=\sqrt{\frac{\mathrm{var}\left(\dot{\xi }\left(t\right)\right)}{\mathrm{var}\left(\xi \left(t\right)\right)}}$$

(10) $$\mathrm{Complexity}:\;C_{\xi }=\frac{M(\dot{\xi }\left(t\right)}{M(\xi \left(t\right)}$$

- Non-stationary index (NSI)

The NSI proposed in [16] measures the complexity of the time series and can capture the non-stationary degree of the signals. The temporal variations of the amplitude of EEG signals are involved, irregular, turbulent, and non-periodic, yet they exhibit long-range correlations over most time scales, indicating the presence of self-invariant and self-similar structures. NSI is easy to implement. It is sufficient to divide the time sequence to be analyzed into several segments, then calculate the local averages of sections, and finally compute the variance of the local averages.

- Fractal dimension (FD)

Another indicator for describing the complexity or chaos of EEG signals is the FD, whose stable and precise values are generally derived through the Higuchi algorithm [17]. Fractal dimension-based features build on evidence that EEG signals can be regarded as a fractal curve, each part of which can be seen as a reduced-scale image of the whole. The calculation procedure is as follows.

First, consider a finite set of time-series observations sampled at a regular interval ξ(t):

(11) {ξ(1),ξ(2),ξ(3),⋯,ξ(T)}

Second, construct a new time sequence, $\xi_{k}^{m}$, from the above-given signal:

(12) $$\begin{array}{c} \left\{\xi \left(m\right),\xi \left(m+k\right),\xi \left(m+2k\right),\cdots ,\xi \left(m+\left[\frac{N-m}{k}\right].k\right)\right\}\\ m=1,2,\cdots ,k \end{array}$$

where [⋅] denotes the Gauss notation for the floor function; *k* denotes the time interval; *m* = 1, 2, ⋯, *k* is the initial time point of each subsequence; both *k* and *m* are integers.

Next, the length of each sub-curve $\xi_{k}^{m}$ is defined as:

(13) $$L_{m}\left(k\right)=\frac{T-1}{k^{2}\cdot \left[\frac{T-m}{k}\right]}\overset{\left[\frac{T-m}{k}\right]}{\underset{i=1}{\sum }}\left|\xi \left(m+ik\right)-\xi \left(m+\left(i-1\right)\cdot k\right)\right|$$

The term $\frac{T-1}{k^{2}\cdot \left[\frac{T-m}{k}\right]}$ is a normalization factor. The sum term represents the sum of the absolute values of the forward difference to the $m_{th}$ set.

Then, the length of the time series or the curve for the time interval k, 〈L(k)〉, is defined as the mean over k sets of $L_{m}\left(k\right)$. Last, if there is $\langle L\left(k\right)\rangle \propto k^{-D_{\xi }}$, then the EEG curve is fractal with the dimension $D_{\xi }$. Note that the FD has not been utilized on periphery physiological channels.

- Higher-order crossing (HOC)

**Figure 4:**
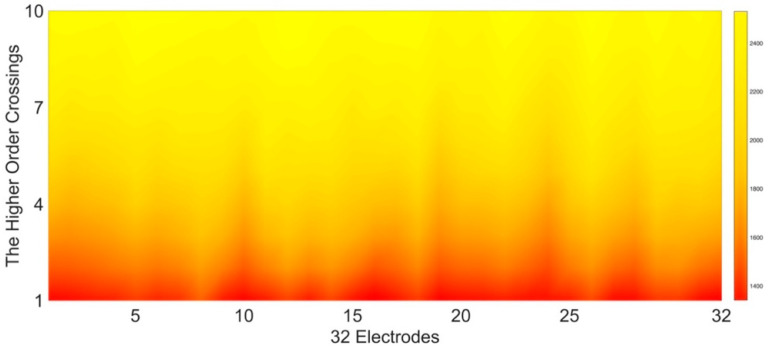
Illustration of the HOC sequence.

Higher-order statistics are found to be more effective. The zero-crossings count of a finitely long real-valued zero-mean series can account for the oscillating attitude around the zero levels, the values of which will consequently vary if a filter is applied to the time series. Naturally, a specific sequence of filters applied to a time series will result in specific zero-crossings counts, which are the so-called HOC-based features that can be viewed as a measure of oscillation pattern in the manner that the stronger the oscillation, the higher the zero-crossing rate, and vice versa. Herein, enlightened by [18], we apply a sequence of high-pass filters based on the backward difference operator to the zero-mean time series ξ(t). 

(14) $$\zeta_{k}\left\{\xi \left(t\right)\right\}=\nabla^{k-1}\xi \left(t\right)$$

where $\nabla^{k}$ is then iteratively applied backward to the difference operator, and k=1, 2, ⋯, 10 is the order. The resulting k features, i.e., $D_{k}$, consist of the zero-crossings count of the filtered time series $\zeta_{k}\left\{\xi \left(t\right)\right\}$. As shown in Figure 4, the values of ten HOC features of the 32 EEG channels are presented in a heat map, and the lighter the color, the larger the value. As the order increases, the zero-crossing count decreases. Higher-order crossings can also apply to the periphery signals.

#### 2.3.2. Frequency Domain

- Power–law index

Signal processing has always relied on power spectral density (PSD) analysis as a conventional instrument for analyzing time series. In particular, when the power spectrum follows the power-law $P\left(f\right)\propto {\left(f\right)}^{-\eta }$, where P(f) denotes the power spectrum density and f in hertz, then the exponent η can also be considered as the index for characterizing the irregularity of signals. It is formally defined as the Fourier Transform (FT) of the autocorrelation sequence (the Wiener–Khintchine theorem, which assumes the stationarity of stochastic signals):

(15) $$P_{\xi \xi }\left(f\right)=\overset{+\infty }{\underset{m=-\infty }{\sum }}R_{\xi \xi }\left(m\right)\exp\left(-j2\pi fm\right)$$

where $R_{\xi \xi }:=E\left[\xi^{*}\left(n\right)\xi \left(n+m\right)\right]$ is the autocorrelation sequence. Since ξ in this article is definitely real-valued, and its autocorrelation is summable (a sufficient but necessary condition for the power spectrum), the power spectrum exists and is real-valued, nonnegative, and symmetrical.

**Figure 5:**
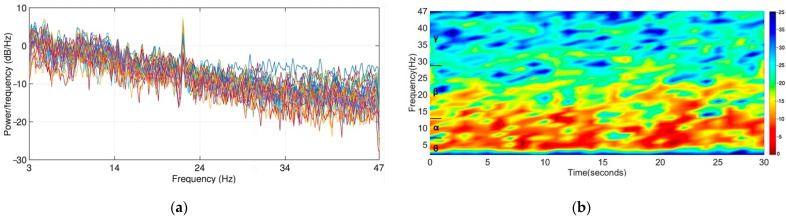
Illustration of the power spectral density of 32 EEG channels and the time–frequency spectrum of an EEG channel derived from the Short-Time Fourier Transform: (**a**) power spectral density; (**b**) the time–frequency spectrum.

To estimate the PSDs of power signals that do not satisfy the generalized stationary assumption, we may use Welch’s averaged, modified periodogram [19]. First, chop a length N signal into length ***L*** overlapping segments of equal length ***M***, i.e., ***N*** = ***L*** **∗ *M***; then window each slice and apply Fourier transform to determine the frequency components at that slice; finally, let the power spectrum of the entire signal by the average overall pieces of data. Herein, an 853-point FFT with a 426-point (half of the window) overlapped Hamming window function w was adopted to generate the PSDs. An example of the power spectral density of EEG signals is illustrated in Figure 5a. The power spectral density curves show a mild trend of the long tail.

- Band Power

Power in four major frequency bands: θ(3–7Hz), α(8–13Hz), β(14–29Hz), and γ(30–47Hz) are the most popular features in EEG-based emotion recognition. Short-Time Fourier Transform is more commonly used to calculate the approximate average power of each band because it is more robust to noise than Fourier Transform, and Figure 5b shows the spectrum of an EEG channel in 30 s at 347 Hz. As one can see, the energy is mainly concentrated in θ and α bands in this case. Here, we adopt the Hamming window with length of 1000 ms with no overlap. The final computed features comprise the logarithm of the average, the logarithm, of the maximum and minimum power of each band, the power variance across all frequency bands, and the average power ratio of β over α.

Spectrogram, the most basic method, relies on conventional Fourier spectral analysis, which assumes piecewise stationarity of the data. The typical implementation of the spectrogram is the Short-Time Fourier Transform (STFT), with which a time–frequency distribution can be obtained by successively sliding the window along the time axis and performing the Fast Fourier Transform on each time window. The uncertainty principle in the field of time and frequency analysis demonstrates that increased time resolution reduces frequency resolution and vice versa. STFT suffers from inaccurate time–frequency positioning due to this conflicting phenomenon. Therefore, it will only serve to compute the band power as described above and will not be further discussed.

- Higher-order spectra (HOS)

The higher-order spectral analysis is a non-linear signal processing framework, building on the fact that much more information can be obtained from an autocorrelation or power spectrum in a stochastic non-Gaussian signal. Higher-order spectra defined in terms of a signal’s higher-order moments or cumulants contain this additional information. The higher-order spectral analysis assumes that the processes or signals of interest are zero means, just as higher-order crossings in the time domain because higher-order cumulants are invariant to mean shifts. Higher-order spectra include polyspectra, such as the bispectrum, bicoherence, trispectrum, etc. Here, we have only analyzed the bispectrum for simplicity. For an in-depth discussion of the definitions of “polyspectrum” and “cumulant” and their properties as well as implementation issues, the reader is encouraged to consult the Higher-Order Spectral-Analysis Toolbox [20].

Bispectrum serves to extract the phase information and characterize the properties of non-linear mechanisms such as quadratic and cubic patterns [21]. The features of the bispectrum were derived for the quantification of emotions in [2]. The bispectrum is defined as the Fourier Transform of the third-order cumulant sequence:

(16) $$S_{3\xi }\left(f_{1},f_{2}\right)=\overset{+\infty }{\underset{k,l=-\infty }{\sum }}C_{3\xi }\left(k,l\right)e^{-j2\pi \left(f_{1}k+f_{2}l\right)}$$

Note that the bispectrum is a function of two frequencies. Herein, the 128 × 128 bispectra are estimated by firstly segmenting the signal into 30 nonoverlapping 128-sample slices (only the second half’s recordings are used), next applying lag-domain windows to the computed cumulants in the nonredundant region, and finally computing a two-dimensional 128-point FFT of the windowed cumulants with shifting and rotating for proper orientation.

**Figure 6:**
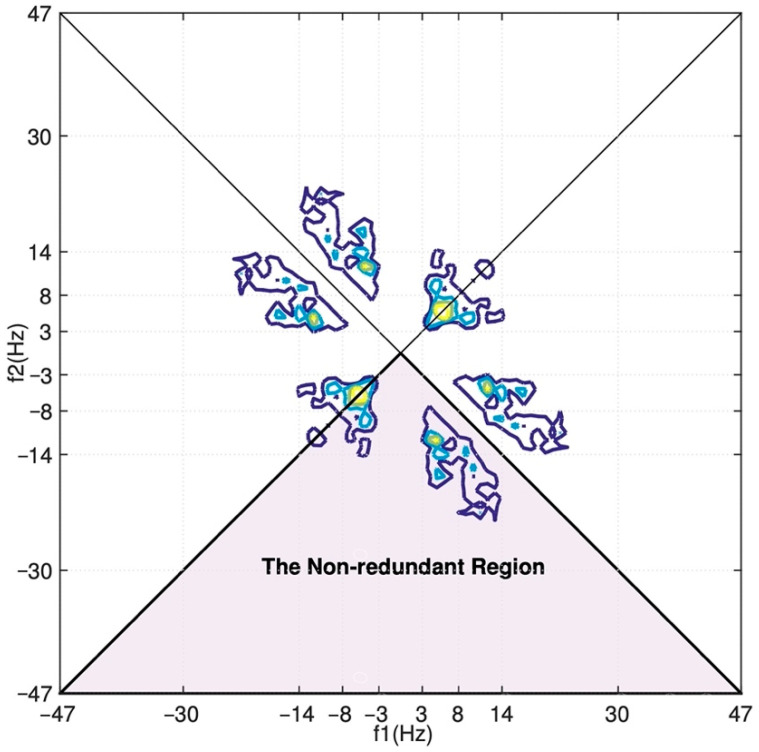
Illustration of the contour plot of bispectrum of an EEG channel.

Due to the vast volume (128 × 128 = 16,384) of the computed bispectrum, we turn to derive the four features proposed in [2] from the bispectrum data:

  1. Normalized bispectral entropy
    (17) $$BE1=-\overset{}{\underset{n}{\sum }}p_{n}\log p_{n},\;p_{n}=\frac{\left|B(f_{1},f_{2})\right|}{\sum_{\Omega }|B(f_{1},f_{2})|}$$
    where Ω is the non-redundant or principal region, as illustrated in Figure 6. For a specific definition of the non-redundant region, the reader is referred to [20,21,22].
  2. Normalized bispectral squared entropy
    (18) $$BE2=-\underset{n}{\sum }q_{n}\log q_{n},\;q_{n}=\frac{{\left|B\left(f_{1},f_{2}\right)\right|}^{2}}{\sum_{\Omega }{\left|B\left(f_{1},f_{2}\right)\right|}^{2}}$$
  3. Mean-magnitude of bispectrum
    (19) $$MMOB=\frac{1}{L}\underset{\Omega }{\sum }\left|B\left(f_{1},f_{2}\right)\right|$$
    where ***L*** is the number of points within Ω.
  4. First order spectral moment
    (20) $$FOSM=\overset{N}{\underset{k=1}{\sum }}\log\left|B\left(f_{1},f_{2}\right)\right|$$
    where *N* denotes the total number of elements in the bispectrum matrix.

- Magnitude-Squared Coherence Estimate (MSCE)

The quantities mentioned above are all computed based on the recordings of one single electrode (except for the average power ratio of β over α). Yet, researchers have found that some electrodes in different scalp regions have a joint reaction while experiencing different affective states [1]. MSCE features describe the correspondence of two signals coming from different channels $\xi_{i}$ and $\xi_{j}$ at corresponding frequency, taking values between 0 and 1 [15]. The MSCE is given in Equation (21):

(21) $$C_{ij}\left(f\right)=\frac{{\left|P_{ij}\left(f\right)\right|}^{2}}{P_{i}\left(f\right)P_{j}\left(f\right)}$$

where $C_{ij}\left(f\right)$ is the MSCE of $\xi_{i}$; $\xi_{j}$ indicates how well $\xi_{i}$ corresponds to $\xi_{j}$ at each frequency; $P_{ij}$ is the cross-power spectral density; $P_{i}$ and $P_{j}$ denote the power spectral density of $\xi_{i}$ and $\xi_{j}$, respectively.

- Differential asymmetry and rational asymmetry

According to neuroscientific findings concerning hemispheric asymmetry and its relationship to emotion [23], features based on the combination of symmetrical pairs of electrodes have garnered attention. They are typically classified as differential asymmetry and rational asymmetry between the electrode pairs symmetrically distributed on the cerebral cortex in the international 10–20 system. The former one usually refers to the average power difference of electrode pairs on the left/right hemisphere of the scalp: $\Delta x=x_{l}-x_{r}$, and the latter refers to the ratio of the pairs: $r_{x}=x_{l}/x_{r}$.

#### 2.3.3. Time–Frequency Domain

It is not new to decompose a signal into its harmonic components. As early as 1677, Newton had carried out the spectral decomposition of light. The Fourier Transform led to modern signal analysis based on transform domain decomposition. The below three methods are designed to modify the global representation of Fourier analysis, but they have all failed in one way or another. In time–frequency analysis, signals are typically decomposed into a small number of components based on the local characteristic time scale. Accurate localization of frequencies is possible. The primary concern is the instantaneous frequency and energy rather than the global frequency and energy defined by Fourier spectral analysis. Time–frequency methods can provide additional information by considering the dynamic changes provided by the non-stationarity of signals, and presenting them as an energy–frequency–time distribution.

- Hilbert–Huang spectrum (HHS)

Using the Hilbert–Huang spectrum, one can localize any event in the time domain and on the frequency axis. The Hilbert–Huang spectrum can be derived by applying the Empirical Mode Decomposition (EMD) function and the Hilbert Transform (HT) function. With the EMD function, any complicated non-linear and non-stationary signal can be decomposed into a series of “intrinsic mode functions” that admit well-behaved Hilbert Transforms. Through the subsequent Hilbert Transform of Intrinsic Mode Functions (IMFs), which are derived from local properties of signals, the instantaneous frequencies and energy are obtained as time functions, resulting in a full energy–frequency–time distribution of the original data. The paper [24] summarized the necessary conditions to represent a nonlinear and non-stationary time series as completeness, orthogonality, locality, and adaptivity, in which completeness guarantees the precision in the expansion and orthogonality avoids leakage, locality serves nonstationary signals that have no time scale, and adaptivity is crucial for avoiding harmonic distortion of non-linear phenomena.

An IMF component involves only one oscillatory mode so that the Hilbert Transform can provide the full description of the frequency content. Contrary to the other expansion methods, the basis of HHS has derived from the raw data of the trait of riding waves expressed as one undulation riding on top of another, and they, in turn, are riding on still other fluctuations so on [24]. The sifting process of systematically extracting the IMFs is expanded in detail, as shown in Algorithm 1. The local maxima and local minima separately define the upper and lower envelopes.
**Algorithm 1.** Hilbert–Huang spectral analysis [24]**Input:** The original signal vector x.**Output:**The Hilbert–Huang spectrum, i.e., an energy-time–frequency distribution of x.1.**function** EMD (x ,SegLen$,\;\mathrm{ResidueThreshold},\;SD_{T})$2.    IMF←03.    i←04.    N←length(x)/SegLen5.    residue←∞6.    while residue>*ResidueThreshold* **do**7.        i←i+18.$\;\;\;\;\;\;\;\;x_{i}\leftarrow x-\sum_{i}IMF$9.        SD←∞10.$\;\;\;\;\;\;\;\;\mathrm{while}\;SD>SD_{T}\;\mathrm{do}$11.            for j=1→j=N **do**12.$\;\;\;\;\;\;\;\;\;\;\;\;\;\;\;\;seg_{ij}\leftarrow x_{i}\left[\left(1+\left(j-1\right)*SegLen\right):\;\left(j*SegLen\right)\right]$13.$\;\;\;\;\;\;\;\;\;\;\;\;\;\;\;\;\left[LocalMax_{ij},\;IndMax_{ij}\right]\leftarrow max\left(seg_{ij}\right)$14.$\;\;\;\;\;\;\;\;\;\;\;\;\;\;\;\;IndMax_{ij}\leftarrow IndMax_{ij}+\left(j-1\right)*SegLen$15.$\;\;\;\;\;\;\;\;\;\;\;\;\;\;\;\;\left[LocalMin_{ij},\;IndMin_{ij}\right]\leftarrow min\left(seg_{ij}\right)$16.$\;\;\;\;\;\;\;\;\;\;\;\;\;\;\;\;IndMin_{ij}\leftarrow IndMin_{ij}+\left(j-1\right)*SegLen$17.                  **end for**18.$\;\;\;\;\;\;\;\;\;\;\;\;\;\;\;\;\mathrm{UpperEn}v_{i}\leftarrow \mathrm{spline}\left(IndMax_{i},\;LocalMax_{i},\;1:length\left(x_{i}\right)\right)$19.$\;\;\;\;\;\;\;\;\;\;\;\;\;\;\;\;\mathrm{LowerEn}v_{i}\leftarrow \mathrm{spline}\left(IndMin_{i},\;LocalMin_{i},\;1:length\left(x_{i}\right)\right)$20.$\;\;\;\;\;\;\;\;\;\;\;\;\;\;\;\;LocalMeanApprox_{i}\leftarrow \left(UpperEnv_{i}+LowerEnv_{i}\right)/2$21.$\;\;\;\;\;\;\;\;\;\;\;\;\;\;\;\;x_{i}\leftarrow x_{i}-LocalMeanApprox_{i}$22.$\;\;\;\;\;\;\;\;\;\;\;\;\;\;\;\;SD\leftarrow \sum \left[\frac{{\left(xTemp-x_{i}\right)}^{2}}{xTemp^{2}}\right]$23.             **end while**24.$\;\;\;\;\;\;\;\;\;\;\;\;{\mathrm{IMF}}_{i}\leftarrow x_{i}$25.$\;\;\;\;\;\;\;\;\;\;\;\;\mathrm{residue}\leftarrow \mathrm{mean}\;(x-\sum_{i}IMF)$26.        **end while**27.    return IMF28.**end function**29. 30.function HT(IMF,fs)31.    zt←hilbert(IMF)32.$\;\;\;\;\mathrm{Energy}\leftarrow {\left(\mathrm{imag}\left(\mathrm{zt}\right)\right)}^{2}+{\left(real\left(\mathrm{zt}\right)\right)}^{2}$33.$\;\;\;\;\mathrm{Phase}\leftarrow arctan\frac{imag\left(\mathrm{zt}\right)}{\mathrm{real}\left(\mathrm{zt}\right)+eps}$34.    frequency←Phase/(2∗π∗fs)35.    **return *energy, frequency***36.**end function**

**Figure 7:**
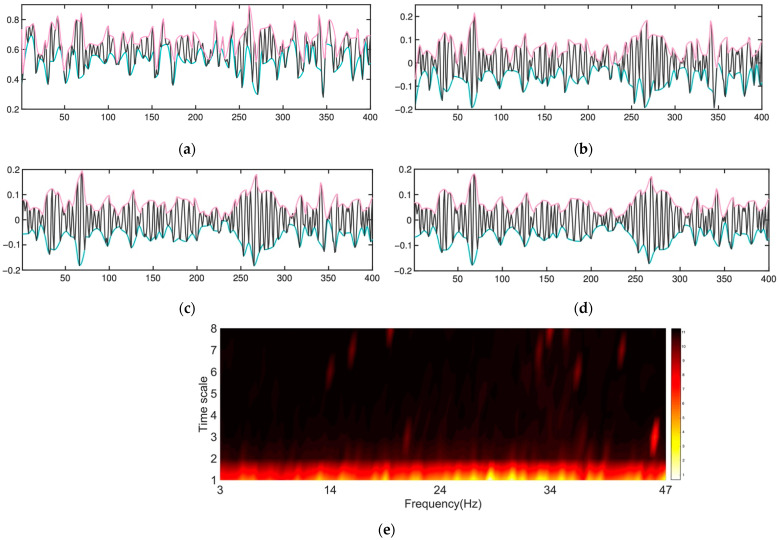
Illustration of the effects of repeatedly applying the sifting operation and the Hilbert–Huang spectrum. (**a**) The original normalized data. The upper and lower bounds are depicted in pink and cyan, respectively. The original data suffers severe asymmetry, the mean of which is not zero. (**b**) After sifting once through the original data. (**c**) After sifting once more, the result is still asymmetric and not an IMF. (**d**) After sifting twice, the result is much better than the original data, but more siftings are needed to mitigate the asymmetry and the ridding waves. (**e**) The Hilbert–Huang spectrum.

As depicted in Figure 7, the first IMF component contains information on the whole frequency range of Hz (3–47), yet the other IMFs include knowledge of much fewer frequencies. For example, the third IMF shows a soft spike at about 21 Hz and an extremely sharp peak at around 44 Hz, and the nineth IMF shows high energy at 23 and 34 Hz.

- Discrete wavelet transform (DWT)

The multi-resolution wavelet approach, gloriously called “the mathematical microscope”, has attracted widespread popularity. It is essentially a linear Fourier spectral analysis with adjustable windows. Unlike the STFT, the wavelet analysis is adaptive to the fluctuations of signals by adjusting the width of the time window and frequency scale. The general continuous definition of WT is:

(22) $$W_{x}\left(a,b\right)=\frac{1}{\sqrt{a}}\int_{-\infty }^{+\infty }x\left(t\right)\psi^{*}\left(\frac{t-b}{a}\right)dt$$

where a is the dilation factor; $\frac{1}{\sqrt{a}}$ gives the frequency scale; b is the translation of the origin and gives the temporal location of an event; ψ(t) is the mother wavelet function. The physical sense of Equation (22) can be interpreted as the energy of x(t) of scale a at time b.

Here, we use the orthogonal and compactly supported the fourth order Daubechies wavelet to decompose the signals into detail coefficients of four levels and approximation coefficients of the fourth level, designated as D1, D2, D3, D4, and A4, respectively. Further, given the sampling rate of preprocessed signals in DEAP is 128 Hz, the corresponding frequency range of each set of coefficients is deduced by the criteria shown in Table 3. The entropy, root mean square (RMS), and the absolute logarithmic recursive energy efficiency (abs(log(REE))) of frequency bands θ,α,β,γ are derived by extending the approach introduced in [22].

#### 2.3.4. Feature Selection for Reducing the Dimensionality

An abundance of features will lead to over-specification, excessive computation load, and even overfitting when some features have little influence on the classification task. Achieving the smallest, most representative, distinguished, and robust feature subset that would yield the minimum generalization error is crucial.

Generally speaking, the feature selection procedure can be considered as an optimization problem for data pre-processing and typically involves two processes. First, a subset search is performed to select a subset via a particular search engine such as the greedy forward, greedy backward, and bidirectional search, among which: the forward search recursively appends relevant features to an initial set; on the contrary, the backward method continually discards irrelevant attributes from a complete feature set; the bidirectional way combines the other two. Besides, some randomized heuristic policies have been used for selection and showed efficiency, too. Second, a subset evaluation is performed to assess the suitability of the candidate subset via the above-mentioned miscellaneous criteria.

There are three general approaches to feature selection: filters, wrappers, and embedded methods. Here, only the first two are considered. Wrapper-type methods judge the availability of a feature by the actual accuracy of the specific classifier, thus guaranteeing a specialized non-redundant feature subset that matches best with the learning method, yet requires extensive computation, so a beforehand filtering is necessary. Filtering methods are model-independent and less computationally intensive, thereby fitting the feet of large datasets. The simplest baseline approach falling in filtering categories is removing features with low variance (RFLV), which is based on the idea that variables with milder undulations can have less impact on the results. The idea is similar to Principal Component Analysis (PCA) motivation, as well as the somewhat controversial Entropy Weight Method.

The relief method inspects the problem from a correlation perspective and chooses the variables with high association with the target. As with Linear Discriminant Analysis, the Fisher score assigns the highest score to features whose values of a different class are far from each other. In contrast, values of the same class are close to one another. One common practice of filtering methods based on mutual information is the Minimal-Redundancy-Maximal-Relevance (mRMR) framework, proposed in [5] to minimize the redundancy in feature sets obtained from the typical methods that rank genes according to some rule and pick the top-ranked ones. Mutual information itself can be used to measure the relevance or similarity of attributes.

### 2.4. Principal Component Analysis Compressed Representations

To examine the performance of manually extracted features, we used a simple and widely used dimension reduction method, principal component analysis, to extract the principal components of the data as reference features in a linear and unsupervised fashion. PCA learns a linear transformation $f\left(x\right)=W^{T}x+b$ of input data $x\in {\mathbb{R}}^{d_{x}}$, in which the columns of the matrix $W\left(d_{x}\times d_{h}\right)$ form an orthogonal basis for the *d*h orthogonal directions. The transformed result is the *d*h uncorrelated components. High-dimensional data can be compressed into a much smaller space while retaining most of the variation in the data set [25]. In many cases, global representation is needed to lessen the calculative burden or visualize samples for better analysis. PCA identifies the principal components or directions in the feature space along which the variance is maximal by the unsupervised combining of all the attributes. The so-called principal components are linear combinations of the original variables. Specifically, the first principal component is the direction in the data points that shows the greatest variation. The second principal component is the direction uncorrelated to the first component and maximizes the variance of samples when projected onto the component. If the data were standardized, then the principal components were the normalized leading eigenvectors of the covariance matrix. In this piece, signals in each channel are represented by the top 25 components whose cumulative contribution rate has reached nearly 100%, instead of 3840 variables to reduce data volume and, in the meantime, minimize information loss. As a result, there are 800 dimensions for EEG and 200 dimensions for the periphery for a trial.

However, the expressive power of linear features learned by PCA is rather limited. One cannot stack these linear features to form deeper representations since the composition of linear operations yields another linear operation.

## 3. Methods

**Figure 8:**
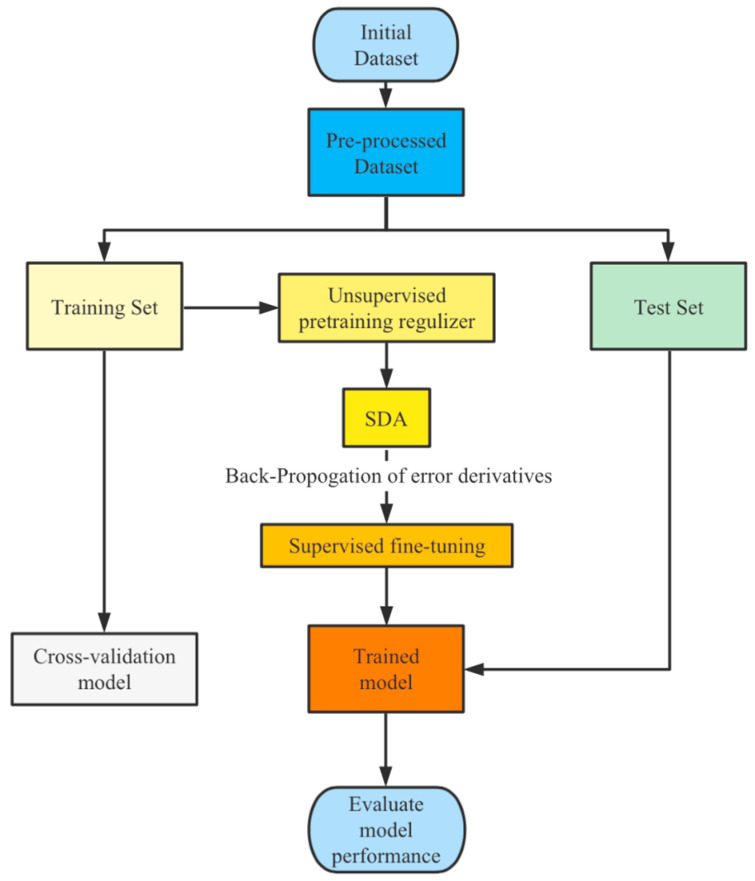
The workflow diagram of the semi-supervised deep-learning architecture.

The success of machine-learning algorithms in pattern recognition heavily relies on data representation as different representations stand for different explanatory factors of variation behind the data [26]. Features have a decisive impact on experimental performance. Designing features based on specific domain knowledge is labor-intensive and contrary to the pursuit of artificial intelligence. Traditional machine-learning algorithms may inherit some critical limitations from features. In contrast, representation-learning algorithms such as probabilistic models, autoencoders, manifold learning, and deep networks can be applied to learn more powerful representations and disentangle the underlying explanatory factors through deep learning whose performance is expected to be comparable to that of humans. They are less dependent on feature engineering. The more abstract and more useful representations are yielded from the composition of multiple non-linear transformations. This paper focuses on the autoencoder model among the various ways of learning representations. This section will present the principles of our semi-supervised method for higher-level feature extraction at length. The workflow diagram of our proposed method is presented in Figure 8.

### 3.1. Stacked Denoising Autoencoder (SDA)

In this section, we will explain the theories and terminologies of autoencoders and denoising autoencoders. We will also explain why we chose to stack an array of denoising autoencoders to reduce the dimensionality of the physiological emotion data.

#### 3.1.1. Traditional Autoencoder (AE)

**Figure 9:**
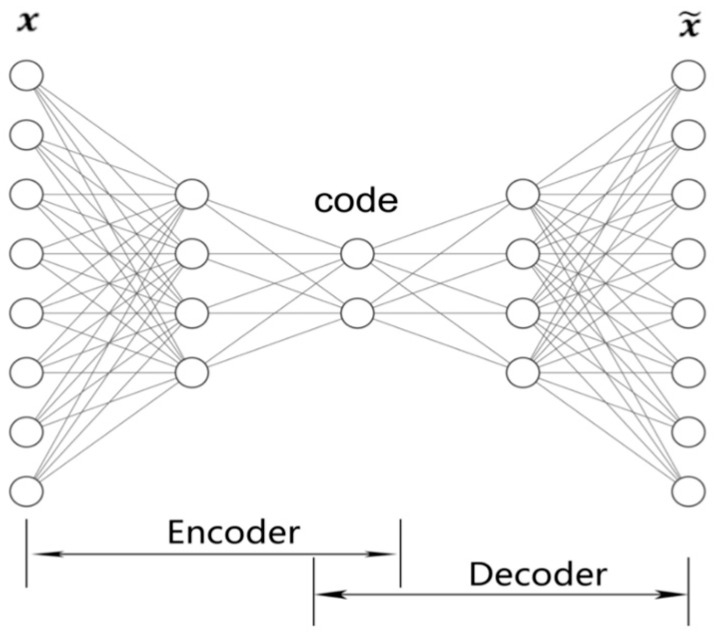
Illustration of a classical two-layer autoencoder. The encoder transforms input data to the central-layer activations, the decoder maps from the feature space back into the input space, producing a reconstruction.

A low-dimensional code can reconstruct high-dimensional input vectors in a small central layer of a neural network. Such networks are called “autoencoder” or “autoassociator” and can work much better than PCA if the initial weights are close to a good solution [27]. Autoencoders can be seen as a non-linear generalization of PCA. An example of the two-layer autoencoder is illustrated in Figure 9, in which the “encoder” network transforms the high-dimensional data into a low-dimensional code and a similar “decoder” network recovers the data from the code. They are trained together by minimizing the discrepancy between the original data and its reconstruction version using Gradient Descent.

One concern and theoretical shortcoming regarding traditional autoencoders is whether it will fail to learn a useful representation when the number of units is not strictly decreasing from one layer to the next. Under such a circumstance, it is theoretically feasible for autoencoders to just learn an identity mapping (simply copying the input, which generally yields zero reconstruction error) under such circumstances with a high capacity. Although Bengio’s [28] experiments had shown that it generalizes well with non-decreasing layers, stacking traditional autoencoders achieved only comparable performance compared with DBN.

#### 3.1.2. Denoising Autoencoder

**Figure 10:**
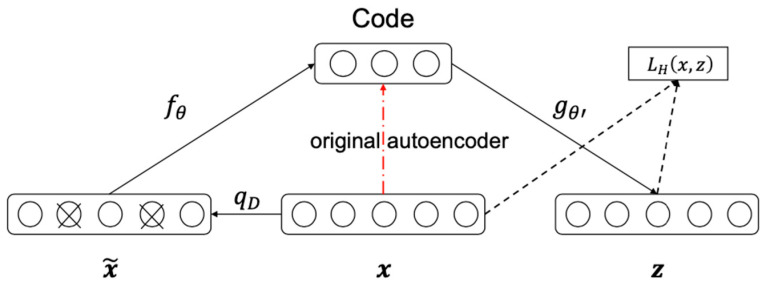
The input x is partially destroyed by some noise $q_{D}$ such as missing values, Gaussian noise, or salt and pepper noise for images, yielding the corrupted input $\tilde{x}$. $\tilde{x}$ is mapped to the hidden code by the encoder function $f_{\theta }$. From the transformed representations, we reconstruct a z by the decoder function $g_{\theta^{\prime }}$. The training criterion is to minimize the reconstruction error $L_{H}\left(x,z\right)$. The classical autoencoder transforms the input directly to the code, as depicted in the red dashed line.

The denoising autoencoder is a straightforward variant of regular autoencoders and is trained to denoise the corrupted version of the input data. As depicted in Figure 10, compared to the original autoencoder, the denoising variant adds a polluting step as expressed in Equation (23), thus making the input of “encoder” the contaminated input data. Such a minor modification has been proven to significantly enhance the autoencoders’ performance.

Here, we calculate the corrupted input $\tilde{x}$ and the reconstructed z as follows:

(23) $$\tilde{x}\leftarrow q_{D}\left(\tilde{x}|x\right)$$

(24) $$h=f_{\theta }\left(\tilde{x}\right)=s_{f}\left(Wx+b\right)$$

(25) $$z=g_{\theta^{\prime }}\left(h\right)=s_{g}\left(W^{\prime }h+b^{\prime }\right)$$

where $f_{\theta }$ and $g_{\theta^{\prime }}$ denote the closed-form parameterized encoder and decoder function, respectively. θ={W,b} and $\theta^{\prime }=\left\{W^{\prime },b^{\prime }\right\}$ are the parameter vectors minimizing the reconstruction error; $b\left(d^{\prime }\times 1\right)$ and $b^{\prime }\left(d\times 1\right)$ are the biases; $W\left(d^{\prime }\times d\right)$ and $W^{\prime }\left(d\times d^{\prime }\right)$ are the weight matrices; h is the computed feature vector; $s_{f}$ and $s_{g}$ denote the encoder and decoder activation function. Typically, the elementwise sigmoid or hyperbolic tangent nonlinearity is chosen for $s_{f}$; if $s_{g}$ is linear, the identity function is set for $s_{g}$.

An intuitive geometric hypothetical interpretation [29] for denoising autoencoders is given by manifold learning, which supposes high-dimensional data concentrate on a low-dimensional manifold. Corrupted examples that are far from the manifold will be projected back to be near the manifold via the training procedure.

#### 3.1.3. Stacked Denoising Autoencoder

Deep architectures can be much more efficient than shallow ones, as highly non-linear and highly varying functions are compactly represented by deep multi-layer neural networks, suggested by the complexity theory of circuits. Stacked denoising autoencoder borrows the greedy principle of DBNs. It is an original way of building deep networks using stacking layers of denoising autoencoders.

Deeper autoencoders can generate lower reconstruction errors than shallower ones [27]. Adding more layers or stacking more one-hidden-layer autoencoders will increase the model’s capacity, enabling the composition of more useful features. In many cases, the denoising criterion that guides the purely unsupervised learning bridges the performance gap of the stacked autoencoder with DBN and even surpasses it in many cases [29]. Higher-level learned representations can boost the performance of subsequent classifiers.

The training procedure of stacked denoising autoencoders can be divided into two subprocesses: the unsupervised pre-training for searching for a proper initialization configuration for parameters in the network and the supervised fine-tuning for moderately adjusting the settings of the parameters. The process can be shown in Figure 8.

### 3.2. Unsupervised Pre-Training

A greedy layer-by-layer unsupervised “pre-training” is an algorithm to find the most appropriate initial configuration of weights and biases. This technique was first proposed by Hinton [30] and extended to cases where input is continuous by Bengio [28]. During the pre-training, layers are trained to represent the dominant factors of the data. Pre-training uses only the underlying knowledge behind the data, not the information contained in the labels, which will be applied in the fine-tuning stage to slightly adjust the weights found by pre-training.

It is believed to be capable of overcoming the challenges of deep learning (as stated in Section 3.1.1) by introducing prior knowledge to the fine-tuning procedure, where a similar argument is found in humans that their capacity to quickly become proficient in new tasks builds on much of what they have learned before facing that task. One reasonable and competing explanatory hypothesis is that the pre-training acts as an unusual regularizer [8]: pre-training would result in a regularization effect by establishing a particular initialization point of the parameter space of fine-tuning and restricting the parameters to be chosen in a relatively small, bounded, and local basin of attraction of the supervised cost function. This type of regularization has precedence in the neural networks in the form of “early stopping.”

The training of ordinary deep architectures is a non-convex optimization problem. However, by initializing weights in a region near a local minimum, pre-training mitigates the complicated non-convex optimization, giving better generalization. Also, pre-training can facilitate the training process of deep networks by significantly reducing the total training time.

### 3.3. Global Fine-Tuning on a Supervised Criterion

For the greedy layer, the driving idea behind semi-supervised learning is to exploit information about the prior probabilities of input data to boost the generation of the classifier. Training cases that belong to the same cluster will be mapped into nearby embeddings.

Pre-training provides a good initialization for supervised fine-tuning of the whole network. The fine-tuning is the second and last stage of training. The parameters of the entire network can be further optimized simultaneously by gradient descent concerning a deterministically computable supervised criterion that depends on the representations [28]. It is worth mentioning that fine-tuning does not change the weights significantly, even with a high number of times of updating.

### 3.4. Deep-Belief Networks

A deep-belief network is another implementation of autoencoders. Pre-training and fine-tuning are still the main form of learning. The layer building block “Restricted Boltzmann Machine” is a bipartite graph modeled by an ensemble of binary vectors, where Gaussian or binary “visible” units are connected to stochastic, binary, and “hidden” feature detectors through symmetrically weighted connections. Restricted Boltzmann Machines (RBM) are a special case of Boltzmann Machine and Markov Random Fields. The joint probability of various values of units in the visible and hidden layer of the RBM is defined by energy:

(26) $$P\left(v,h\right)=\frac{1}{Z}\exp\left(-E\left(v,h\right)\right)$$

where Z is the partition function; v and h represent the states of units in the visible layer and the hidden layer, respectively. If the visible units are binomial, then the energy is calculated as:

(27) $$E\left(v,h\right)=-\underset{i,j}{\sum }v_{i}W_{ij}h_{j}-\underset{j}{\sum }b_{j}h_{j}-\underset{i}{\sum }c_{i}v_{i}$$

where $v_{i}$ and $h_{j}$ are the states of visible neuron i and feature $j;\;b_{i}$ and $b_{j}$ are their biases and $W_{ij}$ is the weight between them. Note that when the input layer units take real values, the neurons of the visible layer follow the polynomial distribution, which is a generalization of the binomial distribution where each unit has multiple possible values.

Block Gibbs sampling determines whether or not the hidden units are activated:

(28) $$\{\begin{array}{c} P\left(h_{j}=1|v\right)=\sigma \left(\underset{i}{\sum }W_{ij}v_{j}+b_{j}\right)\\ P\left(v_{i}=1|h\right)=\sigma \left(\underset{i}{\sum }W_{ij}h_{i}+c_{i}\right) \end{array}$$

where the first layer of feature detectors then become visible neurons for training the next RBM.

Class autoencoders and RBMs are similar in their function form despite the enormous difference in the interpretation and training algorithms. After unsupervised training, an output logistic regression layer is added to estimate the class probabilities using multinomial logistic regression (soft-max).

## 4. Performance and Evaluation

**Figure 11:**
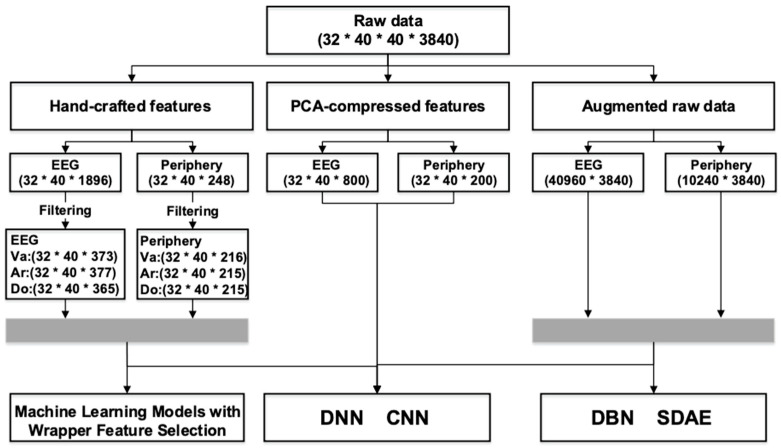
Raw data in DEAP is refined by a bunch of domain-specific algorithms, resulting in 1896 EEG features and 248 periphery features, which are further filtered by hybrid feature selection and taken as input for machine-learning models and the DNN CNN model. To examine the expression ability of hand-engineered representations, we reduce the raw data to its principal components using the widely-employed dimensionality reduction method PCA. The raw data were augmented, normalized, and fed into the SDA model for applying our semi-supervised feature-learning approach. The DBN model is taken as a reference in the meantime.

To investigate and compare the performance of our proposed method with existing mainstream methods that can be summarized as combining one or more human-designed features with one classical learning algorithm, which can be traditional machine-learning methods or deep-learning approaches, we performed substantial experiments in this section, the experimental setup, and the results are elaborated below. All data preparations are detailed in Figure 11.

### 4.1. Hand-Engineered Features

As is exhibited in Figure 11, the number of derived features was huge, which may result in the fact that some features are irrelevant to others. Hence, before proceeding any further, we performed a hybrid filtering selection as a preliminary operation to reduce the dimensionality. The Scikit-learn Python package [31] was used for implementing the machine-learning models.

First, as a pre-step, we utilized the most fundamental RFLV to eliminate a small amount of less volatile features. The threshold was set as 0.01. As a result, a total of 148 EEG features with variances lower than 0.01 were picked out, among which were 18 from the higher-order spectra, 39 from the band power, 21 from the Hilbert–Huang spectrum, 24 from the differential asymmetry, 44 from the rational asymmetry, and 1 from the discrete wavelet transform. As for periphery modality, 31 features were removed, including 20 from the Hilbert–Huang spectrum, 3 from the discrete wavelet transform, 3 from the Hjorth, and 2 from the statistics in the time domain. The results can vary because the threshold was a hyperparameter of the unsupervised RFLV. As a result, the higher-order spectra and Hilbert–Huang spectrum fail to meet the expectation claimed in the literature.

Then, after eliminating features with small variation, we applied two supervised filtering methods and took the union of their choices as the input of learning algorithms with the mode-dependent wrapper selection. The motivation for us to do this was: as a rule, incorporating several state-of-the-art methods of feature selection surpasses a single method in performance, providing a broader coverage of the original space covered by the entire dataset, while emphasizing the discriminative efficiency. To decide which filtering methods to combine, we compared the performance of ten univariate and supervised filtering feature selection methods to recognize the valence dimension.

The result is presented in Table 4. It is clearly exhibited that the chi-square method gets marked (bold or underlined) by four models and wins first place in two of them. The method based on mutual information follows closely behind, being marked for three times and winning a championship twice. Accordingly, filtering based on chi-square and mutual information is incorporated to conduct further filtering after RFLV: the union of the top-ranked 200 features of both methods forms the final feature set for wrapper selection and classification. Specifically, in doing so, we arrive at 373 EEG components and 216 periphery dimensions for Valence, 377 and 215 for Arousal, and 365 and 215 for dominance, as shown in Figure 11.

#### 4.1.1. Using Traditional Machine-Learning Algorithms with Hybrid Feature Selection

To investigate the performance of hand-engineered features and evaluate the contribution of both modalities, we conducted three binary classification tasks using seven widely-used machine-learning models in the literature. As mentioned above, a hybrid strategy of filter and wrapper type methods was employed for feature selection. The final feature subsets for three recognition tasks have been acquired.

Both cross-subject and single-subject classifications are performed. For cross-subject recognition, trials of all 32 participants are mixed, resulting in 1280 pieces of data. The results in Table 5 were obtained through five-fold cross-validation with the greedy forward wrapper search. For single-subject recognition, the 40 trials of one subject formed a tiny individual database. To refrain from overfitting that was prone to small data sets, we performed the leave-one-out-cross-validation for each subject, which can be viewed as 40-fold ordinary cross-validation with only one piece of test data. The accuracy was calculated by dividing the total number of correct testing by 40. In addition, there can be features that can accurately distinguish dimensional size, resulting in 1.0 accuracy by itself. This was due to the small datasets that severely lack training cases. We have skipped such features in the experiment.

**Figure 12:**
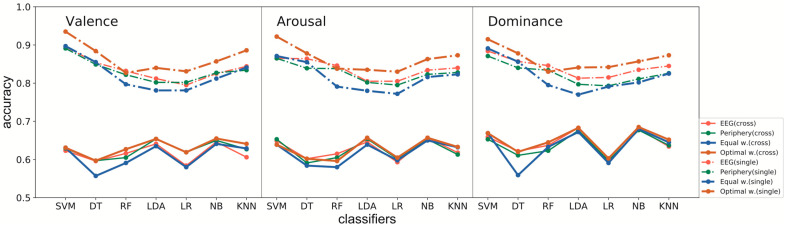
The accuracy of seven traditional learning algorithms for cross-subject and single-subject recognition on three scales is correct. The cross-subject classification tasks are in solid lines; instead, the single-subject ones are in dashed lines. Results using only the EEG modality are in red, green for using the periphery modality alone, blue for decision fusion with equal weights, and brown for decision fusion with the optimal weights found by exhaustive search.

To obtain a better result, decision fusion with equal weights and the optimal weights was also investigated. The exhaustive search found the optimal weights, where the step length was set as 0.01. The fused output was the weighted average of the two sets of output probabilities from EEG and periphery modality. As Table 5 and Figure 12 show, decision fusion with optimal weights outperformed the others, whereas fusion with equal weights showed the poorest performance. In this case, decision fusion using weighted average changes only changed the recognition result that the two modalities disagree on, while optimal weights tended to follow the opinion of the right modality.

In contrast, fusion with equal weights showed no preference, thus making more mistakes. Besides, distinct from the usual declarations in the literature that the periphery is inferior to EEG and plays a supporting role, we observed that the periphery modality drew equally with EEG for the valence scale in both cross-subject and single-subject recognition. For arousal and dominance, although, for the most part, the EEG outperformed the periphery modality, there were times that the periphery matched or beat EEG.

**Figure 13:**
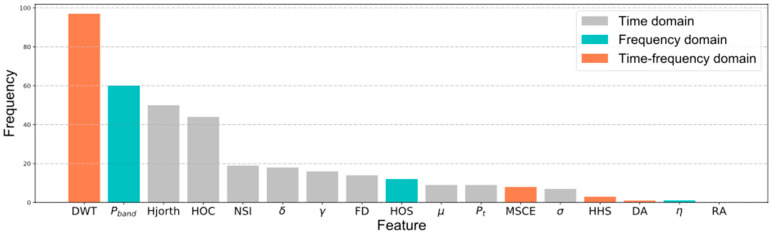
Statistical ordering of all hand-engineered features selected by the machine-learning algorithms for both cross-subject and single-subject recognition on valence, arousal, and dominance dimension.

In addition, the times of various hand-engineered features selected by the machine-learning algorithms for both cross-subject and single-subject recognition on the valence, arousal, and dominance dimensions were added up, as shown in Figure 13. Features derived from the discrete wavelet transform in the time–frequency domain were the most favored. The power of four frequency bands in the frequency domain and the mobility and complexity were defined by Hjorth features in the time domain. Most features in the time domain were running neck-and-neck; however, surprisingly, the Hilbert–Huang spectrum and electrode pairs were not as popular as expected.

#### 4.1.2. Using Deep Neural Networks to Compare with PCA-Compressed Features

To further verify the effectiveness of the well-designed features elaborated in Section 2.3, we will probably use PCA’s oldest feature extraction method as a reference.

We compared the performance of two feature sources: hand-crafted features introduced in Section 2.3 and PCA-compressed features mentioned in Section 2.4. The need for a special note was that only cross-subject recognition were to be performed using deep architectures, and single-cross recognition was merely conducted in Section 4.1.1. In this experiment, all the 1748 features for EEG and 216 for the periphery modality were used without further filtering, except with RFLV. For PCA, the top 25 principal components were retained, so there were 800 components for EEG and 200 for the periphery. Feature fusion by concatenating the two feature vectors is also implemented. A three-hidden-layer deep neural network is used, and the dropout probabilities were set to 0.45, 0.5, and 0.25, respectively. Specifically, we employed a 1748-800-200-20-2 network for hand-engineered EEG, a 217-100-50-10-2 network for the hand-engineered periphery, and a 1965-500-100-20-2 network for the fusion of hand-crafted features. The number of neurons in three hidden layers of PCA were 500-100-20, 100-100-20, and 500-100-20 for the three feature sources. The dataset was divided into a training set with a size of 768, a validation set for adjusting hyperparameters such as learning rate and the number of training epochs, and a testing set with a size of 256. The five-fold cross-validation was also used here.

Our loss function consisted of four terms to avoid overfitting and to reduce the impact of category imbalances:

(29) $$L\left(x\right)=\lambda_{0}*\mathrm{crossenropyerror}+\lambda_{1}*L1+\lambda_{2}*L2+\lambda_{3}*C$$

where $\lambda_{0},\lambda_{1},\lambda_{2},\lambda_{3}$ are the coefficients of the four terms; L1 and L2 denote the L1 regularization loss and the L2 regularization loss, respectively; $C=C_{01}+C_{10}$, C represents the cost of misclassification caused by class imbalance. The four coefficients and other hyperparameters are adjusted according to the crossentropy error.

The results are shown in Table 6. Hand-engineered features outmatch the unsupervised PCA-compressed ones in all the comparisons, demonstrating these human-designed representations’ serviceability and superiority.

### 4.2. Automatically Generated High-Level Representations

This section compares the expressive power of several deep-learning models on an RTX 2060 GPU, including two discriminative models (DNN, CNN) and two generative models (DBN, SDA). The augmented data were utilized as input after normalization.

#### 4.2.1. Supervised Discriminative Architectures

We focused on deep neural networks (DNN) and convolutional neural networks (CNN) for discriminative models. With the popularity of deep learning, DNN and CNN have attracted the most attention. These networks use the original data taken as input directly. Therefore, each layer in these networks can become a special feature extractor without human intervention, similar to the idea of representation learning in generative models. However, generative models can learn more parameters without overfitting, requiring no feedback from the labels. For generative models, the parameters are constrained by the number of bits required to describe the input for each training case. Yet, for discriminant models, the parameters are constrained by only the number of bits needed to describe the label [29]. We ignored the internal differences between the two networks and just used them as examples of discriminative models to compare feature extraction performance with generative models.

**Figure 14:**
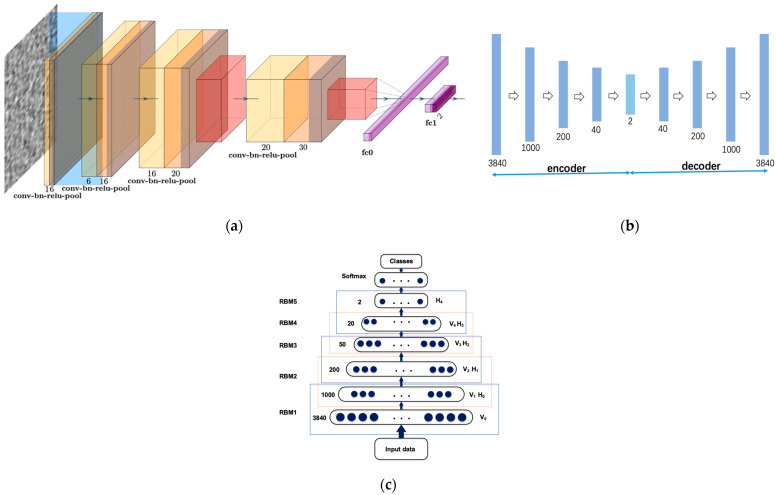
Structures of deep architectures we used in the comparison. (**a**) CNN; (**b**) SDA; (**c**) DBN.

A 3840-800-200-20-2 network was designed for DNN with dropout probabilities set as 0.45, 0.5, and 0.25 for the three hidden layers. Each affine layer was followed by a batch normalization layer and a ReLU activation layer. As illustrated in Figure 14a, the CNN model deployed in the experiment consists of four modules: the first two submodules contain a convolutional layer followed by batch normalization and ReLU; we also added an additional max-pooling layer for the last two modules. The kernel size was set as three, the striding length was set as two, and no padding was applied. A 0.85 dropout probability was set for the fully connected layer. Each piece of data was converted to a 60 × 64 size image.

#### 4.2.2. Semi-Supervised Generative Architectures

Basic concepts of the stacked denoising autoencoder (SDA) and Deep Belief Network (DBN) are similar. The data were first transformed into a new representation by unsupervised pre-training, and the supervised classifier was layered on top to map the representations into class predictions. Indeed, both minimizing the reconstruction error for autoencoders and contrastive divergence training for RBMs can be seen as an approximation of a log-likelihood gradient. However, stacking ordinary autoencoders regularly underperforms stacking-restricted Boltzmann machines. Our method of stacking denoising autoencoders exhibited superior performance in this section.

Considering that our data were real-valued, we used a linear decoder with a squared reconstruction error for SDA, whose structure is shown in Figure 14b. For applying noise to the data, we utilized the standard “dropout” method, i.e., resetting values of a fixed random proportion of original data elements to zero. In particular, the proportion was set at 0.2. The fine-tuning output layer takes input only from the top hidden layer. Once the stack of autoencoders has been trained and the stack of encoders has thus been built, the highest-level output representations would be used as input for a stand-alone supervised learning algorithm. A K-nearest neighbor model and a support vector machine with the radial basis function kernel were implemented as classifiers.

**Figure 15:**
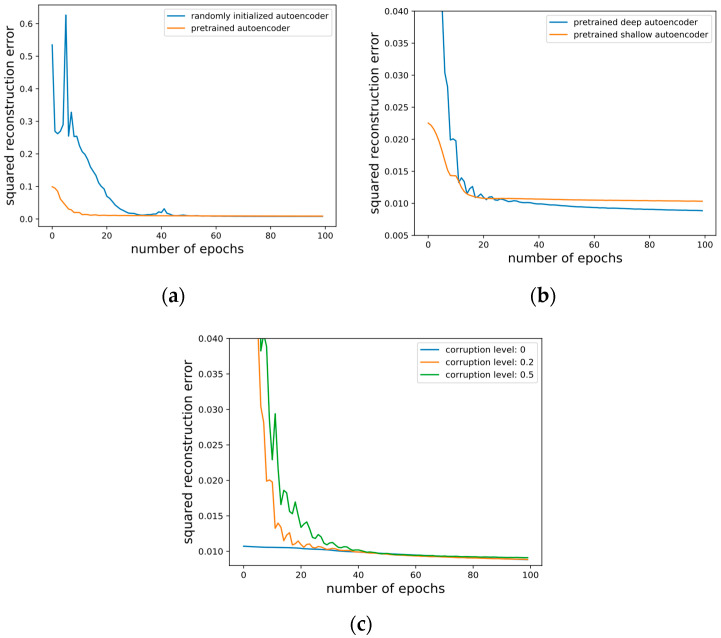
Structures of deep architectures we used in the comparison. (**a**) Pre-training or not; (**b**) depth of the stacking; (**c**) corruption level.

To further investigate the mechanism of action of SDA, we conducted several other experiments. These were to verify the effect of whether to pretrain the depth of stacking and the noise level added in. A 3840-1000-200-40-2 autoencoder was employed. The results are shown in the following Figure 15.

With or without the unsupervised pre-training, it should be pointed out that the supervised optimization objective and the gradient-based scheme are the same. The only thing that differs is the starting point in the parameter space: either randomly selected or obtained by pre-training. The average squared reconstruction error per test data during fine-tuning is shown in Figure 15a. Pre-trained autoencoder has a much lower initial error than those without pre-training. Although both decrease the error to near zero in our task, with or without pre-training, pre-training makes progress faster, thus needing less fine-tuning.

The average squared reconstruction error on the test set is shown in Figure 15b during the fine-tuning. Initially, the error of a deep 3840-1000-200-40-2 autoencoder was greater than that of a the shallower 3840-1000-200-2 autoencoder at first, but the ultimate error of the deeper one went down more rapidly. However, we observed that the hypothesis that the deeper the network, the better the performance does not hold. An appropriate increase in the depth of the network can bring benefits, while an overly large depth seemed to increase the probability of finding poor local minima, resulting in bad performance. A deep 3840-2000-1000-400-100-20-2 autoencoder always got stuck in poor solutions, whereas a shallower 3840-1000-200-40-2 was hardly trapped.

The average squared reconstruction error of different levels of corruption during the fine-tuning on the valence scale using EEG modality only is shown in Figure 15c. A 3840-1000-200-40-2 autoencoder was employed with 200 epochs of pre-training. The larger the noise level, the more prominent the effect of pre-training, and the fewer fine-tunings required.

**Figure 16:**
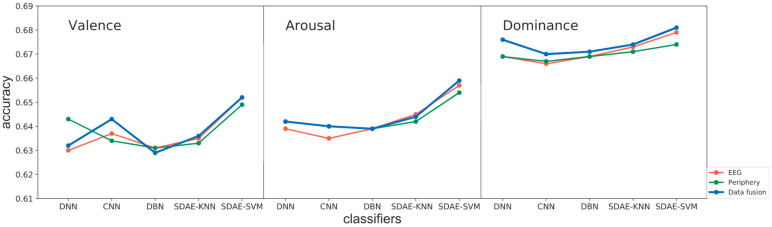
Accuracies of five deep-learning approaches using the augmented raw data as input on three scales of the dimensional effect model. Results of pure EEG and pure periphery are in red and green, respectively; data fusion of both modalities are in blue.

The results of the four deep architectures are shown in Table 7 and Figure 16. The two widely-used discriminative networks and DBN showed similar performances and were slightly defeated by the two SDA models. When it comes to SDA, the SVM shows a slight advantage (shown in bold).

## 5. Conclusions and Future Work

In this paper, we explored learning mapping from input to a novel representation. To circumvent the labor of artificially designed features and leverage the power of deep-learning instruments, we proposed to acquire affective and robust representations automatically through the SDA architecture with unsupervised pretraining, followed by supervised fine-tuning using backpropagation of error derivatives. Performances of the different features and models were compared through three binary classification tasks based on the renowned Valence-Arousal-Dominance affection model. We conducted the following three experiments:

1. Used EEG and periphery signals to generate hand-engineered features.
  (a) We performed decision fusion using traditional machine-learning algorithms with hybrid feature selection;
  (b) We performed feature fusion and used deep neural networks to compare hand-engineered features with PCA-compressed features.
2. Used augmented data as input after normalization.
  (a) We fed the augmented data directly into the SDA (feature extractor) after properly pre-processing and then combined the generated features with the classifier for network fine-tuning. To compare the practical effects of our method, we took two discriminative deep architectures (DNN and CNN) and another generative stacked semi-supervised architecture (DBN) as references.

The results of the mentioned experiments are listed: (1) It turns out that the fusion data achieve performances better than, or in-between the two modalities. Accuracies of single-subject recognition are about 20 percent higher than that of cross-subject classification using well-designed hand-engineered features, demonstrating the “highly-personal” nature of emotion. Besides, statistics from the hybrid feature selection showed that features derived from the wavelet transform and the band power were the most preferred in emotion-recognition tasks. Moreover, the results of the feature fusion of hand-crafted features were 0.651 in valence; (2) Results reveal that our scheme (using the model SVM) with an accuracy of 0.652 in valence (data fusion) slightly outperformed the other three deep feature extractors, and also surpassed the state-of-the-art of hand-engineered features.

Due to the diversity and complexity of human emotions, there were still many problems in correctly recognizing them by machines. The main limitations of our work were as follows:

1. The resulting accuracy was not that high as the model was relatively easy. We need to construct and optimize deep-learning modes by conducting further model fusion to further improve the results.
2. The system needs to select more effective EGG signal features.
3. Due to the limited size of the dataset DEAP, training cases were lacking. A larger dataset should be applied as we need to optimize raw data augmentation to decrease the error.

Although this paper has achieved relatively good results in the presented research, further research is still needed. The top issue in emotion detection is the size of databases. More powerful representations are expected to be built with a larger scale of unsupervised learning. In the scarcity of labeled data and an abundance of unlabeled data, semi-supervised learning is more practical. The pre-training and fine-tuning scale linearly in time and space with the number of training examples. The unsupervised pre-training regularizer can have a more profound positive effect on generalization when the number of training cases is larger. Hence, in the future, deep denoising autoencoders would be very affectionate for non-linear dimensionality reduction, provided that the datasets are big enough.

Future work should include investigating and understanding semi-supervised approaches where one does not separate between the unsupervised pre-training phase and the supervised fine-tuning phase. Such techniques fall more squarely into the field of “semi-supervised” learning. Other avenues for future work include using other types of noise or corruption, and the role of noise level also deserves further inquiry.

It is believed that in the near future, research on emotion-recognition technology in the field of artificial intelligence will make greater progress and be better applied in practical products.

## Figures and Tables

**Table 1:** Comparison of the emotion-recognition methods.

Reference	Dataset	Preprocessing	Feature Extraction	Model	Accuracy
[10]	DEAP	DEAP default preprocessing	GP-LVM: An unsupervised dimension reduction method is suitable for processing small sample and high dimensional data	SVM	VA Medium
[11]	DEAP	DEAP default preprocessing	Divide 8094 datapoints into 10 batches. Nine features including: mean, median, maximum, minimum, standard deviation, variance, value range, skewness, and kurtosis.	DNN; CNN	VA High
[12]	Own dataset		Cepstral coefficients	A novel generalized mixture distribution model	Happy, sad, neutral, and boredom High
[13]	Own dataset: HR-EEG		Nine features: θ, α, β, low γ, and high γ bands, followed by band powers, statistics, and spectral moments	Artificial Neural Network (ANN)	High
This work	DEAP	Feature selection: fusion of removing features with low variance (RFLV) and two wrappers	Seventeen features: discrete wavelet transform (DWT), power band Hjorth, higher-order crossing (HOC), statistics of signals (including 5 features), non-stationary index (NSI), fractal dimension (FD), higher-order spectra (HOS), magnitude-squared coherence estimate (MSCE), Hilbert–Huang spectrum (HHS), differential asymmetry, and rational asymmetry	SVM DT Random Forest (RF) LDA LR Naive Bayes (NB) KNN CNN DNN SDA (feature extractor)	VDA Medium Outperforms other networks

**Table 2:** Summary of extracted features elaborated in Section 2.3 in the light of the domain.

Domain	Features
Time	Power, mean, standard deviation, normalized 1st difference, normalized 2nd difference, mobility, complexity, non-stationary index, higher-order crossings of each channel, fractal dimension for each EEG channel.
Frequency	Power–law index, averaged band power of four frequency bands, BE1, BE2, MMOB, FOSM derived from the bispectrum of each EEG channel; magnitude squared coherence estimate, differential asymmetry, and rational asymmetry averaged on four frequency bands of 14 electrode pairs.
Time–frequency	Entropy and the absolute logarithmic recursive energy efficiency of detail coefficients at four decomposition levels, root mean square of the energy of four frequency bands; averaged energy of four frequency bands of the Hilbert–Huang spectrum of each channel.

**Table 3:** Frequencies corresponding to different decomposition levels for the “db4” wavelet with a sampling frequency of 128 Hz.

Decomposition Levels	Criterion	Frequency Range (Hz)
A4	$0∼\frac{f_{s}}{2j+1}$	0–4
D4	$\frac{f_{r}}{2j+1}∼\frac{f_{s}}{2j}$	4–8(θ)
D3		8–16(α)
D2		16–32(β)
D1		32–64(γ)

**Table 4:** Comparison results of ten notable filtering-type feature selection schemes on several models for the valence scale are given. Accuracies of the top-two methods of each learning algorithm are in bold, and the best ones for each model are underlined besides.

Valence	SVM	DT	RF	LDA	LR	NB	KNN	Mean	Variance
Chi-square	**0.686**	**0.612**	**0.653**	**0.529**	0.611	0.597	0.641	0.618	0.0021
Fisher	0.675	0.589	**0.645**	0.507	**0.631**	0.625	0.641	0.616	0.0026
Gini	0.684	0.601	0.607	0.496	0.584	0.576	0.640	0.598	0.0029
Laplace	**0.690**	**0.620**	0.627	0.509	0.631	0.596	0.640	0.616	0.0026
mRMR	0.671	0.580	0.612	0.510	0.570	0.582	0.607	0.590	0.0020
Pearson	0.680	0.577	0.634	0.523	0.595	0.622	0.643	0.611	0.0022
Relief	0.670	0.580	0.631	0.506	0.616	**0.656**	0.624	0.612	0.0026
Spec	0.671	0.593	0.631	**0.527**	0.606	**0.642**	0.613	0.612	0.0018
Trace	0.682	0.602	0.635	0.520	0.619	0.626	**0.642**	0.618	0.0021
Mutual	0.673	**0.612**	0.641	0.515	**0.634**	0.624	**0.648**	0.621	0.0022

**Table 5:** Accuracies of seven widely used conventional machine-learning models for cross-subject and single-subject recognition on three scales of valence, arousal, and dominance. The best result is shown in bold for each trial, and the more prominent one between the modality of EEG and periphery is underlined. Here, w stands for weight.

Accuracy		SVM		DT		RF		LDA		LR		NB		KNN	
		Cross	Single	Cross	Single	Cross	Single	Cross	Single	Cross	Single	Cross	Single	Cross	Single
Va.	EEG	0.623	0.895	0.596	0.854	0.616	0.832	0.641	0.812	0.584	0.795	0.646	0.825	0.606	0.844
	Periphery	0.629	0.891	0.597	0.849	0.605	0.822	0.654	0.802	0.619	0.802	0.650	0.827	0.627	0.834
	Equal w.	0.597	0.589	0.557	0.855	0.591	0.797	0.635	0.781	0.58	0.781	0.641	0.812	0.629	0.841
	Optimal w.	**0.635**	**0.630**	**0.597**	**0.884**	**0.627**	**0.827**	**0.654**	**0.840**	**0.619**	**0.831**	**0.655**	**0.857**	**0.641**	**0.886**
Ar.	EEG	0.649	0.865	0.602	0.864	0.615	0.846	0.646	0.805	0.593	0.805	0.657	0.834	0.618	0.840
	Periphery	0.653	0.865	0.591	0.839	0.605	0.838	0.654	0.802	0.602	0.795	0.653	0.823	0.613	0.828
	Equal w.	0.721	0.703	0.584	0.855	0.580	0.791	0.639	0.780	0.598	0.772	0.650	0.816	0.632	0.823
	Optimal w.	**0.722**	**0.728**	**0.602**	**0.878**	**0.596**	**0.838**	**0.657**	**0.835**	**0.605**	**0.830**	**0.657**	**0.863**	**0.633**	**0.873**
Do.	EEG	0.659	0.884	0.622	0.857	0.637	0.846	0.683	0.813	0.592	0.815	0.680	0.835	0.634	0.845
	Periphery	0.653	0.871	0.611	0.840	0.623	0.834	0.676	0.797	0.597	0.793	0.677	0.811	0.637	0.826
	Equal w.	0.701	0.702	0.559	0.856	0.633	0.795	0.672	0.770	0.591	0.791	0.680	0.802	0.645	0.825
	Optimal w.	**0.715**	**0.728**	**0.620**	**0.878**	**0.645**	**0.830**	**0.683**	**0.841**	**0.603**	**0.842**	**0.685**	**0.857**	**0.652**	**0.873**

**Table 6:** Performances of hand-engineered features and PCA-compressed features. The higher value of the two features in each modality for each scale is in bold.

Accuracy	Hand-Crafted			PCA-Compressed		
	EEG	Periphery	Feature Fusion	EEG	Periphery	Feature Fusion
Va.	**0.641**	**0.644**	**0.651**	0.639	0.630	0.640
Ar.	**0.694**	**0.644**	**0.653**	0.639	0.639	0.641
Do.	**0.679**	**0.679**	**0.682**	0.669	0.667	0.668

**Table 7:** Accuracies of several deep-learning methods using the augmented raw data.

Accuracy		DNN	CNN	DBN	SDA-KNN	SDA-SVM
Va.	EEG	0.630	0.637	0.631	0.635	**0.652**
	Periphery	0.643	0.634	0.631	0.633	**0.649**
	Data fusion	0.632	0.643	0.629	0.636	**0.652**
Ar.	EEG	0.639	0.635	0.639	0.645	**0.657**
	Periphery	0.642	0.640	0.639	0.642	**0.654**
	Data fusion	0.642	0.640	0.639	0.644	**0.659**
Do.	EEG	0.669	0.666	0.669	0.673	**0.679**
	Periphery	0.669	0.667	0.669	0.671	**0.674**
	Data fusion	0.676	0.670	0.671	0.675	**0.681**

## Data Availability

Not applicable.
